# INSIGHT: A population-scale COVID-19 testing strategy combining point-of-care diagnosis with centralized high-throughput sequencing

**DOI:** 10.1126/sciadv.abe5054

**Published:** 2021-02-12

**Authors:** Qianxin Wu, Chenqu Suo, Tom Brown, Tengyao Wang, Sarah A. Teichmann, Andrew R. Bassett

**Affiliations:** 1 Wellcome Sanger Institute, Wellcome Genome Campus, Hinxton, Cambridge CB10 1SA, UK.; 2 Department of Paediatrics, Cambridge University Hospitals, Hills Road, Cambridge CB2 0QQ, UK.; 3 Department of Chemistry, University of Oxford, Chemistry Research Laboratory, 12 Mansfield Road, Oxford OX1 3TA, UK.; 4 Department of Statistical Science, University College London, 1-19 Torrington Place, London WC1E 7HB, UK.; 5 Department of Physics/Cavendish Laboratory, University of Cambridge, JJ Thomson Ave., Cambridge CB3 0HE, UK.

## Abstract

We present INSIGHT [isothermal NASBA (nucleic acid sequence–based amplification) sequencing–based high-throughput test], a two-stage coronavirus disease 2019 testing strategy, using a barcoded isothermal NASBA reaction. It combines point-of-care diagnosis with next-generation sequencing, aiming to achieve population-scale testing. Stage 1 allows a quick decentralized readout for early isolation of presymptomatic or asymptomatic patients. It gives results within 1 to 2 hours, using either fluorescence detection or a lateral flow readout, while simultaneously incorporating sample-specific barcodes. The same reaction products from potentially hundreds of thousands of samples can then be pooled and used in a highly multiplexed sequencing–based assay in stage 2. This second stage confirms the near-patient testing results and facilitates centralized data collection. The 95% limit of detection is <50 copies of viral RNA per reaction. INSIGHT is suitable for further development into a rapid home-based, point-of-care assay and is potentially scalable to the population level.

## Teaser

INSIGHT is a two-stage COVID-19 test, which combines point-of-care diagnosis with NGS, aiming to achieve population-scale testing.

## INTRODUCTION

The coronavirus disease 2019 (COVID-19) pandemic is caused by the severe acute respiratory syndrome coronavirus 2 (SARS-CoV-2) virus (*1*). Pandemic control has been challenging because of the long incubation period and high percentage of asymptomatic carriers of this disease (*2*, *3*). Nucleic acid testing is thus essential to identify and isolate infected individuals at an early stage to stop the spread of the virus. At the moment, the mainstream nucleic acid test relies on a reverse transcription polymerase chain reaction (RT-PCR) assay, performed on nasopharyngeal and/or oropharyngeal swabs (*4*). It requires labor-intensive RNA extraction and expensive equipment such as a thermocycler. The complexity, cost, and availability of RNA extraction kits and thermocyclers have limited the throughput of these RT-PCR assays. Hence, although the current testing regime, in conjunction with the lockdown measures, has successfully brought down the reproduction number *R* of the disease to below 1 in many countries, ramping up testing capacity sufficiently to maintain *R* below 1 will be challenging once social activities return to normal. At the same time, a prolonged lockdown is highly detrimental to the economy and the physical and mental health of individuals. Regular, high-throughput testing with rapid results is one way out of the current conundrum. Several point-of-care diagnostic tests have been proposed, and some already authorized for use around the world, including SAMBA II (*5*), Abbott ID NOW (*6*), and many others. However, they typically require relatively expensive instruments or reagents, thus limiting their widespread adoption at a population level.

**Fig. 1:**
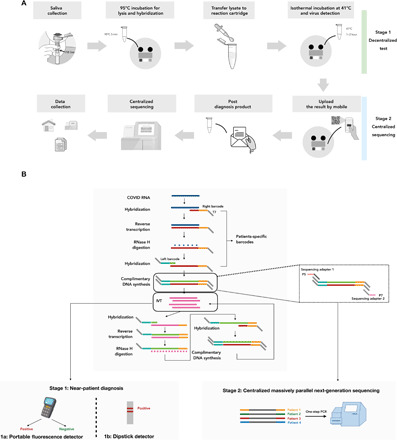
INSIGHT: an all-in-one platform for two-stage COVID-19 detection. (**A**) Schematic outline of our proposed two-stage testing strategy. Stage 1 is a rapid, portable decentralized COVID-19 test. Saliva can be collected directly into a tube containing QuickExtract lysis buffer at home or point of care. After heating up to 95°C to lyse the virus and inactivate proteinase K, the lysate can be transferred into the NASBA reaction and incubated at 41°C. After 1 to 2 hours, the result can be directly visualized with a portable fluorescence detector or lateral flow assay. In stage 2, individuals can post the used reaction mixture to a local sequencing center. All samples are pooled and sequenced after a one-step universal PCR. (**B**) Barcoded NASBA with dual readout for COVID-19 detection. In the NASBA reaction, a sample-specific left barcode is inserted between the sequencing adaptor and the forward primer, and a right barcode is inserted between the T7 promoter and the reverse primer. The NASBA in vitro transcribed RNA product can be used to carry out either a portable fluorescence detection assay or a dipstick-based lateral flow assay for rapid results. Afterward, the NASBA products can be pooled and sequenced. RNase H, ribonuclease H; IVT, in vitro transcription.

The ideal test would have the following five features: It would be accurate, cheap, scalable, portable, and fast. This would allow for decentralized and frequent testing of a large proportion of the population, even in countries with limited medical resources. Numerous groups are working on near-patient tests (*7*–*13*) aimed at improving testing capacity and ultimately achieving regular populational scale testing. However, it is difficult to control for patient operational error, and it is also challenging for centralized data collection. Centralized testing using a next-generation sequencing (NGS) readout (*14*) has also been proposed, which allows efficient scaling of testing and simple data collection, but patients do not have immediate access to the testing results, thus delaying the early isolation of presymptomatic or asymptomatic patients. Here, we propose INSIGHT [isothermal NASBA (nucleic acid sequence–based amplification) sequencing–based high-throughput test], a two-stage testing strategy, using a combination of isothermal NASBA and NGS technologies, combining the advantages of near-patient and centralized testing (Fig. 1A). The first stage of INSIGHT is the NASBA reaction, which can generate rapid test results on the spot in 1 to 2 hours. The second stage uses NGS to improve the test accuracy in a highly scalable way. Below, we describe the two stages of INSIGHT in detail.

The first stage consists of an isothermal NASBA reaction (Fig. 1B) with crude saliva as sample input that could be incorporated into a point-of-care or home-based kit. NASBA uses reverse transcription and T7 RNA polymerase–mediated in vitro transcription to rapidly amplify RNA of interest. More than a billion-fold amplification can be typically achieved in less than 2 hours (*15*). Compared to RT-PCR, the isothermal nature of NASBA means that special equipment, such as thermocyclers, are not needed. NASBA reactions amplify both RNA and DNA, providing unique advantages for the dual-stage diagnosis. For the rapid testing stage, the single-stranded RNA can bind efficiently to complementary oligonucleotides without prior denaturation. A fluorescent molecular beacon can thus be used as a readout to monitor the amplification in real time and yield rapid test results (*16*). Furthermore, we also established a lateral flow assay for a quick and cheap dipstick-based readout. The second stage uses NGS to further improve the test accuracy and reduce user errors in a highly scalable manner. To achieve multiplexed sequencing, a sample-specific barcode pair can be incorporated into the amplified sequence (amplicon) during the first-stage reaction. The stage one end product can then be sent to a central facility for pooled sequencing, allowing up to hundreds of thousands of samples to be analyzed on a single next-generation sequencer. Here, the DNA in the NASBA end product is more stable and less susceptible than the RNA to degradation during the sample shipment process. NGS may also substantially reduce any possible false-negative or false-positive results.

Furthermore, the INSIGHT technology can be viewed as a modular system, with the first stage consisting of two rapid test modules (either fluorescence or dipstick based) and the second stage a sequencing module. These modules can be used alone or combined in different ways, making INSIGHT highly flexible to adapt to different testing needs and resource availability. For example, for areas without adequate sequencing facilities, the rapid test modules (fluorescence or lateral flow based) in stage 1 could be used as standalone tests. In other cases, where accessing NGS is not a limiting factor and quick assay turnaround time could be achieved by well-established logistics and NGS infrastructure, the NASBA reaction with sequencing (stage 2) could be applied alone, reducing the need for fluorescence detectors or purchasing the lateral flow/dipstick consumables. In an ideal situation, both stage 1 and stage 2 could be combined, providing the benefits of both rapid and scalable diagnosis as well as centralized validation and data collection.

## RESULTS

### INSIGHT technology development and optimization

Primers were designed to target the SARS-CoV-2 S gene, which encodes the viral envelope spike glycoprotein. The S gene is one of the most highly expressed viral RNAs (*17*) and, at the same time, is a promising target for SARS-CoV-2 vaccine development (*18*). In addition, the S gene sequence is an informative sentinel for viral genome evolution with respect to increased or decreased affinity to human viral entry receptors such as angiotensin-converting enzyme 2 (ACE2) (*19*). We screened 13 pairs of primers and selected the most efficient pair to optimize our in-house NASBA reaction (fig. S1A). All subsequent reactions and figures shown in this paper were carried out with primer pair P8 (see Materials and Methods). We have performed a homology search using nucleotide BLAST (*20*) and did not find significant homology between the P8 primers and any sequence present in a list of common respiratory microorganisms (list attached in table S2). With synthetic SARS-CoV-2 RNA as the input, we have achieved target RNA amplification with both our in-house NASBA mixture and a commercial ready-made mixture. The NASBA reaction product was confirmed by an RNA urea gel (fig. S1B), showing a product of the expected size in the presence of viral template RNA only. Furthermore, we also optimized the primer concentration used in the NASBA reaction by quantifying the NASBA end product RNA concentration with a molecular beacon (fig. S1C). We found that a primer concentration of 25 nM each, which is 10 times less than typically used in the literature (*21*–*23*), increased the reaction efficiency. Additional optimization has been carried out for our in-house NASBA reaction mixture. This includes the choice of enzymes (ProtoScript II reverse transcriptase instead of avian myeloblastosis virus reverse transcriptase; fig. S1D), the concentration of enzymes (higher concentration with better yield and more consistent results; fig. S1E), and the buffer pH (fig. S1F).

To work toward an INSIGHT test that can be used at home and to simplify the assay workflow and improve its scalability, the NASBA reaction would ideally be applied to a crude saliva lysate. This would alleviate the need for complex and expensive processes to purify RNA. Commercially acquired human saliva can be mixed with QuickExtract buffer and heat-treated at 95°C for 5 min to generate saliva lysate (*24*). Here, we demonstrate that saliva lysate is compatible with the NASBA reaction using commercially available human saliva from healthy individuals with spiked-in synthetic viral RNA. To minimize the handling steps, saliva lysate is combined with partial NASBA mix (without the enzyme cocktail) and heated at 95°C for 5 min to inactivate proteinase K and disrupt RNA secondary structure (fig. S2A). This suggests that in practice, the 95°C denaturation step could be combined with the 95°C viral lysis step to save an extra heating step. The method performs well, with 100 copies of input RNA reliably detected.

Last, to achieve better assay sensitivity, we also varied saliva lysate input volume to find the maximum compatible saliva input amount. We found that saliva lysate can be increased from 1 to 3 μl in a total reaction volume of 20 μl and the detection threshold was not compromised (fig. S2B).

### INSIGHT stage 1 (option A): Fluorescence readout with molecular beacon detection

We have established two forms of specific readouts for stage 1 of INSIGHT: a molecular beacon with fluorescence detection and a dipstick-based lateral flow assay. We describe the performance of the former option here and the latter in the next subsection.

**Fig. 2:**
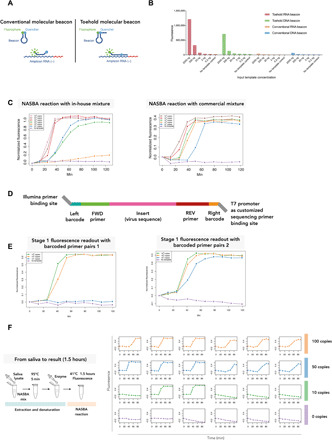
INSIGHT stage 1 fluorescence readout with molecular beacon detection. (**A**) Illustration of conventional and toehold molecular beacons. (**B**) Sensitivity of different types of molecular beacons. Four different types of beacons were mixed with increasing amounts (0.2 to 2000 ng) of in vitro transcribed target amplicon RNA for a fluorescence readout. (**C**) In-house and commercial NASBA mixtures with varying amounts (10^1^ to 10^6^ copies) of input viral RNA templates and a real-time molecular beacon readout. With a final concentration of 20 nM beacon in the reaction, fluorescence was detectable at around 30 min and reached saturation at around 60 min. (**D**) Schematic illustration of the barcoded NASBA DNA product. Using the primer pair P8, the forward (FWD) primer contains a 24-nt sequence targeting part of the SARS-CoV-2 S gene, a 5-nt left barcode, and a 33-nt sequence of the Illumina sequencing adaptor. The reverse (REV) primer contains a 22-nt sequence complementary to part to SARS-CoV-2 S gene, a 5-nt right barcode, and a 31-nt T7 promoter. Customized sequencing primers can be used for the reverse read. (**E**) With barcoded primers, the NASBA reaction achieved a detection threshold of around 10 to 100 copies of viral RNA per 20 μl of reaction for the commercial NASBA mixture (two replicates). (**F**) Left: Schematic illustration of experimental setup with 5 min at 95°C to denature the viral RNA, followed by 1.5 hours at 41°C for the NASBA reaction. Right: Real-time INSIGHT stage 1 fluorescence readings for samples with 0, 10, 50, and 100 copies of viral RNA per 20 μl of reaction (six technical replicates each).

Molecular beacons are hairpin-shaped molecules with a fluorophore and a quencher covalently attached and brought into close proximity due to the secondary structure formed by the hairpin. Upon recognition of the target, the fluorophore and the quencher will be spatially separated because of hybridization, which results in fluorescence (Fig. 2A). Here, four types of beacons were designed to target the P8 amplicon: a conventional DNA beacon, a toehold DNA beacon, a conventional 2′-*O*-methyl RNA beacon, and a toehold 2′-*O*-methyl RNA beacon. The toehold provides an initial anchor point for the beacon to latch onto its target and assists in the unwinding of the stem of the beacon, and the 2′-*O*-methyl modification increases target affinity and provides stability against oligonucleotide degradation. All four types were first tested with in vitro transcribed RNA, and the 2′-*O*-methyl RNA toehold beacon was found to achieve the best sensitivity (Fig. 2B). Therefore, we chose the RNA toehold beacon for our COVID-19 NASBA assay. An essential feature of this beacon is a 3′-propyl group that prevents any possibility of polymerase extension of the toehold sequence.

We have also titrated the concentration of the toehold RNA beacon in the reaction mixture to achieve the best real-time results, as a high beacon concentration inhibits the NASBA reaction (fig. S1G). We found that 20 nM molecular beacon resulted in the best assay sensitivity. With this beacon concentration, the fluorescence reaches a plateau at around 40 to 60 min into the reaction. With the nonbarcoded primer pair P8, the real-time detection limit is around 100 to 1000 copies per reaction for our in-house mixture and around 10 copies per reaction for the commercial mixture (Fig. 2C).

To incorporate patient-specific barcodes in the NASBA reaction, we have designed the primers with 5–nucleotide (nt) barcodes flanked on each side and an Illumina handle on the forward primer (sequences shown in Table 2) so that the NASBA DNA product would contain a 5-nt barcode at each end (Fig. 2D). The NASBA reaction still worked with the barcoded primers, despite a slightly reduced detection threshold of around 10 to 100 copies per reaction for the commercial NASBA mixture (Fig. 2E). By using combinatorial barcoding, a 5-nt barcode sequence on both sides can generate up to a million unique barcodes or 16,384 Hamming distance three-separated barcodes. The latter allows single-nucleotide substitution error in the barcodes to be corrected after sequencing. If needed, then a 6-nt barcode on each primer can generate 262,144 Hamming distance three-separated unique barcode pairs (note S1).

We assessed the performance of INSIGHT stage 1 with real-time fluorescence detection using human saliva from healthy individuals with spiked-in synthetic viral RNA, following temperature setting (2) in step 2 option A in Materials and Methods (Fig. 2F). For samples with input of 50 viral RNA copies per 20 μl of reaction, all six reactions showed amplification of fluorescence signals, whereas for samples with input of 10 viral RNA copies per 20 μl of reaction, two of six reactions showed amplification.

### INSIGHT stage 1 (option B): Dipstick readout with lateral flow assay

We have also developed and optimized a dipstick-based lateral flow assay as an alternative to fluorescence detection in stage 1 of INSIGHT. Compared to molecular beacon detection where a portable fluorescence detector is needed, dipstick-based detection is a particularly attractive option, as it does not require any extra equipment apart from a portable heating source.

**Fig. 3:**
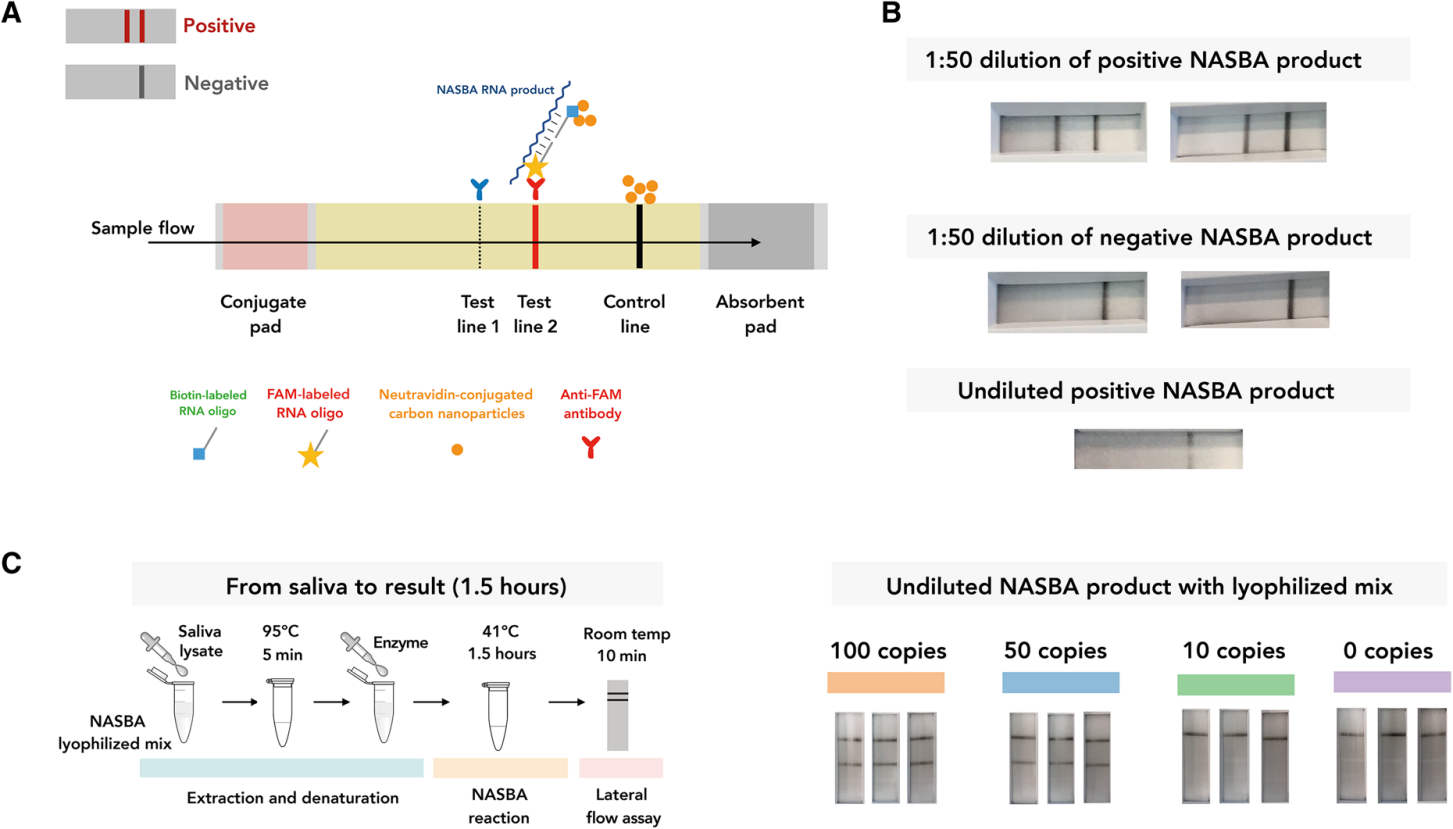
INSIGHT stage 1 dipstick readout with lateral flow assay. (**A**) Schematic illustration of the nucleic acid lateral flow assay. (**B**) Lateral flow–based dipstick test results from NASBA reactions using liquid NASBA mix. The lateral flow assay works when the NASBA end product is diluted 50 times with PCRD buffer but not with undiluted NASBA end product. (**C**) Left: Schematic illustration of experimental setup with 5 min at 95°C to denature the viral RNA, followed by 1.5 hours at 41°C for the NASBA reaction. Lyophilized NASBA mix is used in the reaction. The NASBA end product can be directly loaded onto the dipstick. Results can be read after 10 min of incubation at room temperature. Right: All three technical replicates for 50 copies viral RNA per 20 μl of reaction were detectable using the dipstick assay. Photo credit: Qianxin Wu, Wellcome Sanger Institute.

Here, we use a format of lateral flow assay that detects nucleic acid using neutravidin-conjugated carbon nanoparticles (NA-CNPs) that can bind to biotin, a test line comprising an anti-FAM (fluorescein amidite) antibody and a biotin control line (C-line) to capture excess NA-CNPs. Two RNA capture oligos are added into the NASBA reaction. One is FAM labeled, and the other is biotin labeled. During the reaction, both capture oligos bind to different parts of the single-stranded RNA NASBA product. The dual tagging of FAM and biotin results in aggregation of NA-CNPs at the test line, resulting in a visible signal in the assay (Fig. 3A). A positive lateral flow assay would show a clearly visible line at test line 2 in addition to the C-line, whereas only the C-line is visible in a negative assay. By using the commercially available lyophilized NASBA mix, samples at the end of the NASBA reaction can be directly loaded onto the dipstick without any extra step of RNA purification or dilution (see step 2 option B in Materials and Methods for details).

We also assessed the performance of INSIGHT stage 1 with dipstick detection using human saliva from healthy individuals with spiked-in synthetic viral RNA (Fig. 3C). Three technical repeats were performed for different amounts of viral RNA input. This successfully detected viral RNA with input of 50 copies per 20 μl of reaction and failed to detect samples with input of 10 copies per 20 μl of reaction. We used barcoded primers in the reactions here to make the stage 1 product compatible with stage 2 sequencing.

We note that it is necessary to use the lyophilized mix to achieve a sample-to-dipstick result without any extra steps. We have also tried using the commercially available liquid NASBA mix but found that it required an extra step of dilution before the lateral flow assay (Fig. 3B), possibly due to a component within the liquid mix interfering with the downstream lateral flow assay. In addition, we previously attempted to use biotinylated uridine 5′-triphosphate (UTP) and FAM-tagged RNA capture oligo for dual tagging. However, this combination requires an extra step of RNA purification after the NASBA reaction to prevent unused biotinylated UTP from saturating the NA-CNPs (fig. S3). In summary, we have optimized the conditions such that the lyophilized mix allows direct sample-to-result readout.

### INSIGHT stage 2: Pooled library construction and NGS readout

**Fig. 4:**
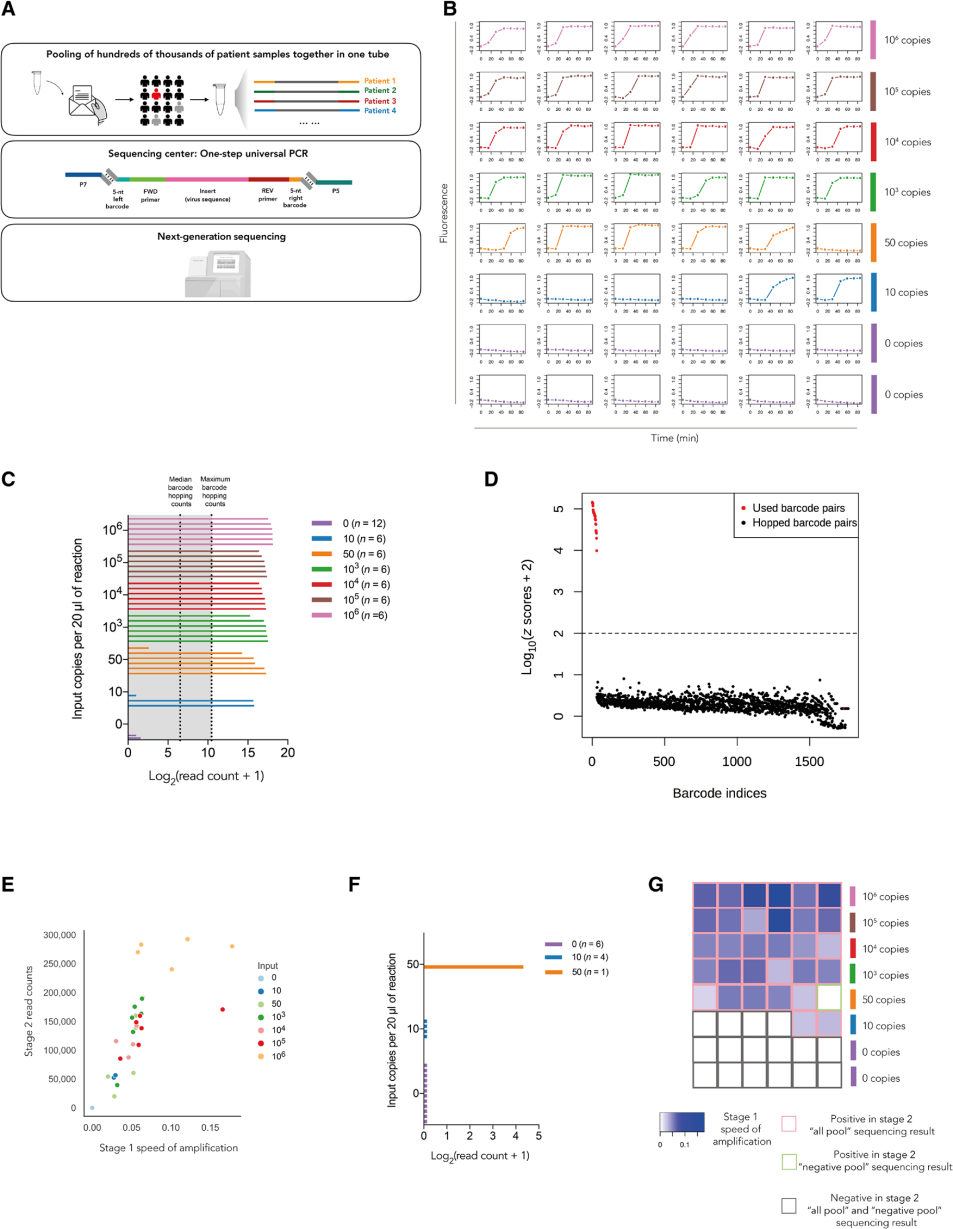
INSIGHT stage 2 pooled testing using NGS. (**A**) Strategy for stage 2 NGS-based detection. Up to hundreds of thousands of samples can be potentially pooled together, and a universal indexing PCR can be carried out at a local sequencing center. (**B**) Real-time molecular beacon readings for 48 barcoded primer pairs with various amounts (0 to 10^6^ copies) of viral RNA input per 20 μl of reaction. (**C**) INSIGHT stage 2 read counts for samples from all reactions in (B) i.e., all pool. The two dotted lines show the median and the maximum of barcode hopping read counts. (**D**) The *z* score distribution of used barcode pairs and hopped barcode pairs using statistical modeling. Red dots, used barcode pairs; black dots, hopped barcode pairs. (**E**) Correlation analysis of INSIGHT stage 1 speed of amplification (reciprocal of time to reach 0.2 normalized fluorescence reading) and stage 2 sequencing read counts. (**F**) INSIGHT stage 2 read counts for samples from 17 reactions in (B) that showed negative results in stage 1 fluorescence detection, i.e., negative pool. All but one samples had 0 count. (**G**) Combined results from both stage 1 and stage 2. The color of the heatmap represents INSIGHT stage 1 speed of amplification with the same sample order in (B). Samples that were positive in INSIGHT stage 2 all pool sequencing results are boxed in red. Samples that were positive in INSIGHT stage 2 negative pool sequencing results are boxed in green. Samples that were negative in both all pool and negative pool sequencing results are boxed in black.

After the NASBA reaction, which can occur in a near-patient setting, we propose that the INSIGHT stage 1 products can be transported to a local sequencing center. As sample-specific barcodes would have already been incorporated in stage 1 in a decentralized manner, all samples can be directly pooled, and the NGS library can be prepared with a simple one-step PCR using primers flanked with sequencing adapters (Fig. 4A and see step 3 in Materials and Methods for details).

We designed an experiment with 48 contrived samples (Fig. 4B). Each reaction used a unique pair of barcoded primers. We varied the input of the viral RNA as shown in Fig. 4B to mimic the wide range of viral load in patient samples. The first stage of INSIGHT was carried out with real-time fluorescence detection. For samples with input of 50 viral RNA copies per 20 μl of reaction, five of six reactions showed amplification of fluorescence signals, whereas for samples with input of 10 viral RNA copies per 20 μl of reaction, two of six reactions showed amplification (Fig. 4B). For the second-stage sequencing, we performed two pooling strategies either independent of the first-stage results or dependent on the first-stage results. “All pool” included all 48 samples regardless of the first-stage results. “Negative pool” was the collection of the 17 samples that showed negative results in the first stage. Library preparation was performed separately for all pool and negative pool.

Figure 4C shows the read counts from all samples in all pool. In addition to reads that contain the expected barcode pairs, we also observed some reads with left and right barcode combinations that were not used in the experiment, a phenomenon we refer to as “barcode hopping.” Among the 17.4 million reads (excluding PhiX reads) generated by MiSeq, 4.96 million reads match the expected amplicon (see step 4 of the “Experimental protocol” section) up to two substitution errors, of which 4.53 million reads contained used barcode pairs and 0.43 million reads were generated from barcode hopping. The two dotted lines in the plot of Fig. 4C indicate the median and the maximum read counts of all hopped barcode pairs. Read counts from samples with 50 viral RNA copies and 10 viral RNA copies showed that five of six and two of six, respectively, had higher read counts than the maximum read count of hopped barcode pairs, providing an identical result to the fluorescent readout. In this particular experiment, both left and right barcodes in every sample are unique, i.e., no two samples share the same left or right barcode. Therefore, hopped barcode pairs can be unambiguously identified and evaluated in this experiment.

To increase the available barcode pair combinations for multiplexing, repeated usage of the same left or right barcode is desirable. We have thus sought to evaluate and potentially circumvent the barcode hopping problem and have built a statistical model (note S2) to predict the number of reads generated from barcode hopping. If the observed read count of a barcode pair is significantly larger than the predicted read count (*z* score exceeding 100), then the barcode pair is likely to be a real product of NASBA amplification, i.e., a positive result. Using the above calling procedure, we can unambiguously identify 31 of the 36 samples with viral RNA input as “positive” and all 12 negative control samples as “negative” in all pool (Fig. 4D). This result is identical to the stage 1 fluorescence result.

We also performed a correlation analysis between the INSIGHT stage 1 speed of signal amplification (measured as the reciprocal of time to reach a normalized fluorescence level of 0.2) and the stage 2 read counts. As expected, there is a strong positive correlation between the results from the two stages (Pearson *R* = 0.67, *P* < 0.0001; see Fig. 4E). Although the input RNA varied from 10 to 10^6^ copies per reaction, the INSIGHT stage 2 read counts for amplified samples only differed by 14-fold. This showed that the NASBA reaction in stage 1 had saturated for most samples, thus allowing stage 2 to handle samples across an extremely wide dynamic range of viral RNA input molecules.

Figure 4F shows the read counts from all samples when sequenced in the negative pool. One sample, which had 50 copies of viral RNA as input and failed to be picked up as positive in stage 1, showed positive read counts in stage 2. This means that stage 2 sequencing can further improve the sensitivity of stage 1 results. INSIGHT stage 1 and stage 2 combined results are summarized and shown in Fig. 4G.

### INSIGHT performance evaluation and comparison with RT quantitative PCR

**Fig. 5:**
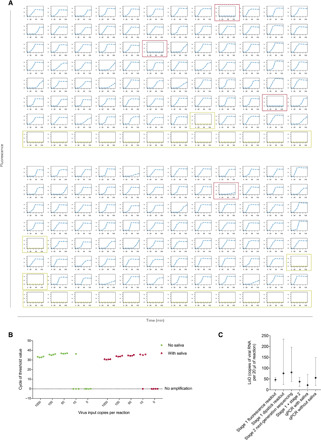
INSIGHT technology LoD and comparison with RT-qPCR. (**A**) Real-time molecular beacon readings for 164 randomly selected barcoded primer pairs with 50 copies of viral RNA input per 20 μl of reaction. Empty wells are boxed in green, and samples that failed to be amplified are boxed in red. (**B**) The qPCR cycle number to reach fluorescence threshold using the Public Health England (PHE) protocol and various numbers of viral RNA copies per 20 μl of reaction as input. (**C**) The estimated 95% LoD (LoD-95) for different stages of INSIGHT and gold-standard RT-qPCR, with the whiskers spanning the 95% CIs of the estimates.

We next sought to summarize the limit of detection (LoD) for the INSIGHT technology. Here, LoD-95 was defined to be the input viral RNA amount at which 95% of samples can be detected. This is estimated by maximum likelihood estimation under the assumption that the number of viral RNA copies in the reaction input follows a Poisson distribution and that each molecule has the same probability of being amplified. INSIGHT stage 1 with a fluorescence readout attains an estimated LoD-95 of 46.6 [95% confidence interval (CI), 37.8 to 56.8] copies per 20 μl of reaction (Fig. 5C). This is calculated on the basis of the 24 reactions shown in Fig. 2F, the 48 reactions shown in Fig. 4B, and additional 327 reactions shown in Fig. 5A and fig. S4. Please note that all barcodes used are randomly selected. The high number of successful amplifications at low copy viral input (50 copies per reaction in Fig. 5A and 100 copies per reaction in fig. S4) indicates that the proportion of the barcodes that might interfere with the NASBA reaction is very low. Separately, INSIGHT stage 1 with dipstick readout has an LoD-95 of 75.8 (95% CI, 24.9 to 234) copies per 20 μl of reaction, which is computed using reactions shown in Fig. 3C. For the second stage, using the all pool results shown in Fig. 4C, we calculated that the NGS sequencing alone has an LoD-95 of 80.3 copies (95% CI, 37.7 to 197) per 20 μl of reaction. By combining the stage 1 fluorescence readout and stage 2 negative pool results, the overall INSIGHT technology LoD-95 can be further improved to 37.8 (95% CI, 16.2 to 57.1).

To put our LoD figures in context, we compared our method with the gold-standard reverse transcription quantitative PCR (RT-qPCR) assay. We performed RT-qPCR (Fig. 5B) following the protocol recommended by the Public Health England (PHE) (*25*). Chemically synthesized viral RNA was used as sample input. Four technical replicates each were carried out with and without saliva lysate from healthy individuals. The RT-qPCR could consistently detect samples with 50 copies of viral RNA per 20 μl of reaction. With 10 copies of viral RNA per 20 μl of reaction, it detected three of four samples with saliva lysate and only one of four without saliva lysate. Thus, the estimated LoD-95 for the RT-qPCR is 55.3 (95% CI, 20.2 to 149) copies per 20 μl of reaction without saliva and 21.4 (95% CI, 7.3 to 70.5) copies per reaction with 1 μl of saliva lysate added in the reaction. In summary, the INSIGHT protocol is highly comparable to the PHE RT-qPCR protocol in terms of sensitivity.

## DISCUSSION

INSIGHT’s dual-stage design combines the benefits of both near-patient testing and centralized testing. It offers a rapid first-stage readout without delay and minimizes the problem of RNA degradation. The distributed first stage also reduces the logistic burden and labor requirements for carrying out population-scale screening. The centralized second stage can be used to eliminate user errors, collate results in a centralized repository, and help inform other epidemiological efforts. In particular, when screening asymptomatic individuals, it is crucial to control false positives, which often requires a confirmatory test for all positive samples. Our second stage here can naturally act as a confirmatory test of near-patient first-stage results. In addition, INSIGHT’s two stages can be viewed as three different modules, two rapid detection modules (fluorescence detection or dipstick-based detection) and one sequencing module. The modules can be either combined in a two-stage test as illustrated here or used independently. This offers flexibility for adaptation to local needs and resources and for testing other viruses.

Our assay sensitivity is comparable to the gold-standard RT-qPCR test. Although INSIGHT has shown promising results in experimental settings, additional work is required to bring it into practical use. The current experiments were carried out using saliva from healthy individuals with spiked-in viral RNA rather than patient samples. However, our estimated LOD-95 of 37.8 copies per reaction (which can have 0.5 to 1.5 μl of saliva input) is well below the median viral load of 7796 copies/μl (interquartile range of 408 to 215,500 copies/μl) in patients’ saliva for the first 7 days from symptom onset (*26*).

In addition, several techniques we developed as part of the INSIGHT technology may be of interest in other contexts. First, we show that toehold beacons can achieve a much higher sensitivity than commonly used regular molecular beacons for fluorescence detection. Second, the use of two RNA capture oligos with different tags in the INSIGHT stage 1 dipstick readout offers a way to detect RNA in a lateral flow assay and further increases specificity for a particular target sequence. Last, we have established a statistical model to address the problem of barcode hopping that can be used in any multiplexed NGS settings.

Other isothermal methods have been proposed for SARS-CoV-2 detection. Most of them are based on the reverse transcription loop-mediated isothermal amplification (RT-LAMP) technology with a colorimetric or turbidimetric readout (*7*–*10*). These assays could potentially suffer from false-positive results generated from nonspecific primer binding or primer dimers (*11*). To circumvent this, Joung *et al.* (*12*) and Broughton *et al.* (*13*) have proposed to use a CRISPR-based assay to achieve highly specific test results. A major advantage of INSIGHT is to enable a rapid readout with high specificity for SARS-CoV-2, while avoiding complex additional steps such as CRISPR-based cleavage. In the first stage, a molecular beacon for fluorescence readout or RNA capture oligos for lateral flow readout provides an additional layer of sequence-specific detection. In the second stage, NGS further improves the assay sensitivity and specificity by unambiguously identifying the viral sequence. We have not observed any false-positive results in all our stage 1 and stage 2 experiments. We note that, unlike the concatemer amplification product in LAMP, the NASBA reaction generates a single, well-defined amplicon, thus making it particularly suitable for sequencing. Furthermore, it allows combinatorial barcoding, thus avoiding the need to synthesize hundreds of thousands of barcoded oligonucleotides.

While different research groups [e.g., (*14*)] and companies [e.g., COVIDSeq (*27*) from Illumina and SwabSeq (*28*) from Octant] have proposed to use NGS to expand testing capacity, most of them are RT-PCR based, restricting amplification and barcoding to centralized facilities. The decentralized first-stage NASBA reaction in INSIGHT greatly reduces the burden of sample handling in testing centers and, hence, makes regular population-scale screening feasible. Other groups including (*11*, *29*–*31*) have also independently suggested similar approaches of barcoded isothermal amplification. We have summarized the main features of a few representative COVID-19 testing technologies, including INSIGHT, in table S3.

Our system can also be modified to include additional primers targeting a different region of the SARS-CoV-2 genome or other pathogens, making it possible to multiplex detection or apply the technology to novel pathogen outbreaks. In addition, an internal positive control RNA sequence that can be amplified by one of the same primer pairs, or a separate primer pair targeting human RNA, can be added to the reaction to ensure that a negative result is not due to faulty reagents or patient handling mistakes and to provide some degree of quantification of viral load. The global COVID-19 emergency has exposed our weaknesses in responding to a new pathogen and an emerging pandemic, and we hope that the INSIGHT two-stage testing strategy has potential to affect this pandemic and beyond.

## MATERIALS AND METHODS

### Experimental protocol

Reagents and oligonucleotides used are listed in Tables 1 and 2. The molecular beacons were synthesized using a K&A H-8 SE DNA synthesizer and purified by reverse-phase high-performance liquid chromatography (ATDBio). Molecular beacons are reconstituted with annealing buffer [10 mM tris (pH 8) with 10 μM MgCl_2_] to the final concentration of 10 μM and then annealed by incubation at 85°C for 5 min and then gradual cooling to 4°C by 0.1°C/s before the NASBA reaction.

**Table 1:** Reagents. DTT, dithiothreitol; DMSO, dimethyl sulfoxide; dNTP, deoxynucleotide triphosphate; NTP, nucleotide triphosphate; BSA, bovine serum albumin; HS, high sensitivity; dsDNA, double-stranded DNA; qPCR, quantitative PCR; NEB, New England Biolabs.

**Reagent**	**Source**	**Identifier**
Twist synthetic SARS-CoV-2 RNA control	Twist Bioscience	Mt007544.1
QuickExtract DNA Extraction Solution	Lucigen	QE09050
NASBA liquid kit	Life Sciences Advanced Technologies Inc.	SKU: NWK-1
NASBA-lyophilized kit	Life Sciences Advanced Technologies Inc.	SKU: NLK
Tris (1 M) (pH 8), RNase free	Invitrogen	AM9855G
Sodium hydroxide	Sigma-Aldrich	71687
1 M MgCl_2_	Invitrogen	AM9530G
2 M KCl	Invitrogen	AM9640G
DTT	Sigma-Aldrich	43816
DMSO	Sigma-Aldrich	276855
dNTP set (100 mM)	Invitrogen	10297018
NTP set (100 mM)	Thermo Fisher Scientific	R0481
RNase H	NEB	M0297L
ProtoScript II reverse transcriptase	NEB	M0368S
T7 RNA polymerase	NEB	M0251L
BSA (20 mg/ml)	NEB	B9000S
PCRD lateral flow immunoassay	Abingdon Health	FG-FD51673
Qubit RNA HS Assay Kit	Invitrogen	Q32852
Qubit dsDNA HS Assay Kit	Invitrogen	Q32851
PowerUp SYBR Green qPCR Master Mix	Applied Biosystems	15340939
Normal human saliva	MyBioSource	MBS170210
QIAquick PCR Purification Kit	QIAGEN	28104
KAPA HiFi HotStart ReadyMix PCR Kit	Kapa Biosystems	KK2600
AMPure XP	Beckman Coulter	A63882
MiSeq Reagent Kits v2 (300 cycles)	Illumina	MS-102-2002

**Table 2:** Oligonucleotide sequences.

**NASBA primers (P8)**	
Forward primer	CCAGCAACTGTTTGTGGACCTA
Reverse primer with T7 handle	aattctaatacgactcactatagggagaaggACACCTGTGCCTGTTAAACCAT
Forward primer with 5-nt barcode and Illumina handle	tgactggagttcagacgtgtgctcttccgatctnnnnnCCAGCAACTGTTTGTGGACCTA
Reverse primer with 5-nt barcode and T7 handle	aattctaatacgactcactatagggagaaggnnnnnACACCTGTGCCTGTTAAACCAT
**Toehold molecular beacon (2′-*O*-methyl RNA)**	
FAM-AUUGACAGUCUACUAAUUUGGUUAAAAACAAAUGUGUCAA-BHQ1dT-UUCAACUUCAAUG-propyl	
**P8 RNA capture oligos for PCRD**	
Probe A	FAM-**AAAAGUCUACUAAUUUGGUUAAAA**
Probe B	**ACAAAUGUGUCAAUUUCAACUUCA**-Biotin
**Library construction PCR primers**	
P5 end primer	AATGATACGGCGACCACCGAGATCTACACNNNNNNNNAGCCAGCTCTGGAGAATTCTAATACGACTCACTATAGGGAGAAGG
P7 end primer	CAAGCAGAAGACGGCATACGAGATNNNNNNNNGTGACTGGAGTTCAGACGTGTGCTCTTCCGATCT
Customized NGS primer (T7 containing)	AGCCAGCTCTGGAGAATTCTAATACGACTCACTATAGGGAGAAGG

#### Step 1: Lysis of saliva samples

Mix crude saliva (commercial pooled human saliva from healthy individuals) at 1:1 ratio with QuickExtract DNA Extraction Solution. Incubate at 95°C for 5 min to ensure complete lysis of virus and inactivation of proteinase K.

#### Step 2 (option A): NASBA reaction with fluorescence detection

Take 1 μl from the product of step 1 (saliva lysate) and add into the NASBA reaction mixture (without the enzyme mix) to make a total volume of 15 μl. Reaction mixture can either be prepared in-house or from the Life Sciences NASBA liquid kit (see Table 3 below) using one of the two temperature settings below.

1) Reaction mixture without the enzyme mix is incubated at 65°C for 2 min, followed by a 10-min incubation at 41°C. Following that, 5 μl of enzyme mix is added into the reaction and incubated at 41°C for a further of 90 to 120 min.

2) Alternatively, reaction mixture without the enzyme mix is incubated at 95°C for 5 min, followed by a 10-min incubation at 41°C. Following incubation, 5 μl of enzyme mix is added into the reaction and incubated at 41°C for a further 90 to 120 min.

**Table 3:** INSIGHT reaction mixtures.

**Life Sciences reaction mixture (RM)**			
	**Volume**	**Stock concenctration**	**Concentration in RM**
Saliva lysate	1 μl		
Primers/beacon mix	1 μl	500 nM each primer	25 nM each primer
		400 nM beacon	20 nM beacon
Spiked-in viral RNA/water	3 μl		
Buffer (NECB-24)	6.7 μl		
Nucleotide (NECN-24)	3.3 μl		
Enzyme mix (NEC-1-24)	5 μl		
**Total volume**	20 μl		
**In-house reaction mixture**			
	**Volume**	**Stock concentration**	**Concentration in RM**
Saliva lysate	1 μl		
Primers/beacon mix	1 μl	500 nM each primer	25 nM each primer
		400 nM beacon	20 nM beacon
Spiked-in viral RNA/water	4 μl		
Buffer with DMSO*	5 μl		
Nucleotide mix*	4 μl		
Enzyme mix*	5 μl		
**Total volume**	20 μl		

*See table S1 for detailed composition.

A fluorescence plate reader (e.g., FLUOstar) can be used to monitor the reaction in real-time or as an end point assay.

#### Step 2 (option B): NASBA reaction with lateral flow dipstick detection

For detection with a lateral flow assay, a NASBA-lyophilized kit is used with the constitution of the reaction mixture shown in Table 4. Take 4 μl from the product of step 1 (saliva lysate) and add into the NASBA reaction mixture (without the enzyme mix) to make a total volume of 60μl. Incubate at 95°C for 5 min, followed by a 10-min incubation at 41°C.

**Table 4:** Lateral flow reaction mixture.

	**Volume**	**Stock** **concentration**	**Concentration** **in RM**
Saliva lysate	4 μl		
Primers	4 μl	500 nM each primer	25 nM each primer
Spiked-in viral RNA/ water	8.16 μl		
Probe A	1.92 μl	1 μM	24 nM
Probe B	1.92 μl	1 μM	24 nM
Reconstituted lyophilized reaction buffer mix (LRB)	40 μl		
			
Reconstituted lyophilized enzyme mix (LEM)	20 μl		
**Total volume**	80 μl		

Following that, 20 μl of enzyme mix is added into the reaction and incubated at 41°C for a further of 90 to 120 min. Take the reaction product to the sample well of a PCRD test cassette. Results will be shown within 10 min.

#### Step 3: Library construction for NGS

To allow for pooled sequencing of NASBA reaction end products, barcode sequences are added upstream of each of the forward and reverse primers (Fig. 4A). In addition, an Illumina sequencing adaptor is added upstream of the forward primer barcode sequence as a universal PCR handle (see Table 2 for the oligonucleotide sequences).

NASBA end products (2 μl) from each sample are first pooled into a single tube. Pooled products are then column purified to remove residual NASBA primers (QIAquick PCR Purification Kit). PCR is performed on the column-purified pooled sample using two NGS indexing primers and the reaction mix and cycling parameters in Table 5. Here, we have designed a customized NGS primer containing the T7 polymerase promoter sequence (see Table 2 for the oligonucleotide sequence) at the P5 end and used a standard TruSeq sequencing primer at the P7 side. A PCR mix is made on the basis of Table 5 below. A standard PCR program is used with longer elongation time and minimal cycle number to reduce barcode hopping.

**Table 5:** Library construction PCR conditions.

**Reaction mixture**		
		**Volume**
2× PCR mix (KAPA HiFi HotStart ReadyMix)		20 μl
P5 end primer (10 μM)		1 μl
P7 end primer (10 μM)		1 μl
Column-purified NASBA product (~3.5 ng dsDNA)		4 μl
Nuclease-free water		14 μl
**Thermocycling conditions**		
**Temperature**	**Time**	**Cycle number**
95°C	3 min	1
98°C	20 s	15
60°C	15 s	
72°C	30 s	
72°C	4 min	1

After the PCR, an AMPure bead–based double size selection is carried out (0.55× and 0.75×) to enrich for products of interest. In this study, NGS was carried out using 150–bp (base pair) paired-end MiSeq sequencing with MiSeq Reagent Kit v2 (300 cycles). Before sequencing, 30% PhiX was added into the library to increase the complexity.

#### Step 4: Analysis of NGS results

To analyze the INSIGHT NGS data, sequences in FASTQ files are first trimmed to leave the first 80 nucleotides for both read 1 and read 2 using FASTX_trimmer. The trimmed read 1 and paired read 2 are then merged by FLASH. The merged sequence is compared with the 102-nt reference viral genome sequence (NNNNNACACCTGTGCCTGTTAAACCATTGAAGTTGAAATTGACACATTTGTTTTTAACCAAATTAGTAGACTTTTTAGGTCCACAAACAGTTGCTGGNNNNN, where N stands for the barcode position), and only those with a Hamming distance of less than or equal to 2 are extracted. Here, only substitutions were allowed, while insertion- and deletion-containing reads were filtered out. The first 5-nt and the final 5-nt regions of all extracted sequences correspond, respectively, to the right barcode and the reverse complement of the left barcode. Diagnostic results for sequenced NASBA samples are determined according to the read counts of their corresponding sample-specific barcode pairs (only sequences with exact barcode match were counted). More details can be found in Results.

### Ethical statement

In accordance with guidance obtained from the Human Tissue Authority, there was no requirement to seek ethics committee approval for the use of commercially acquired human saliva, as test validation did not constitute research because it was determined to be “performance assessment” and sample donor consent was not required.

## SUPPLEMENTARY MATERIALS

Supplementary material for this article is available at http://advances.sciencemag.org/cgi/content/full/7/7/eabe5054/DC1

## Appendix

View/request a protocol for this paper from *Bio-protocol*.
